# Mitochondria Encoded Non-coding RNAs in Cell Physiology

**DOI:** 10.3389/fcell.2021.713729

**Published:** 2021-07-30

**Authors:** Xu Liu, Ge Shan

**Affiliations:** Hefei National Laboratory for Physical Sciences at Microscale, The CAS Key Laboratory of Innate Immunity and Chronic Disease, Division of Life Science and Medicine, Department of Clinical Laboratory, The First Affiliated Hospital of USTC, School of Basic Medical Sciences, University of Science and Technology of China, Hefei, China

**Keywords:** mitochondria, mitochondria-encoded non-coding RNA, lncRNA, dsRNA, small ncRNA, circRNA

## Abstract

Mitochondria are the powerhouses of mammalian cells, which participate in series of metabolic processes and cellular events. Mitochondria have their own genomes, and it is generally acknowledged that human mitochondrial genome encodes 13 proteins, 2 rRNAs and 22 tRNAs. However, the complexity of mitochondria derived transcripts is just starting to be envisaged. Currently, there are at least 8 lncRNAs, some dsRNAs, various small RNAs, and hundreds of circRNAs known to be generated from mitochondrial genome. These non-coding RNAs either translocate into cytosol/nucleus or reside in mitochondria to play various biological functions. Here we present an overview of regulatory non-coding RNAs encoded by the mammalian mitochondria genome. For overall understandings of non-coding RNAs in mitochondrial function, a brief summarization of nuclear-encoded non-coding RNAs in mitochondria is also included. We discuss about roles of these non-coding RNAs in cellular physiology and the communication between mitochondria and the nucleus.

## Introduction

Mitochondria are vital to cells and are involved in multiple essential cellular events. On top of generating ATP, mitochondria also produce reactive oxygen species (ROS), redox molecules, and intermediates required for the synthesis of biomolecules. Furthermore, they are key hubs of intracellular signaling pathways and participate in response to external environmental changes (McBride et al., 2006).

**FIGURE 1:**
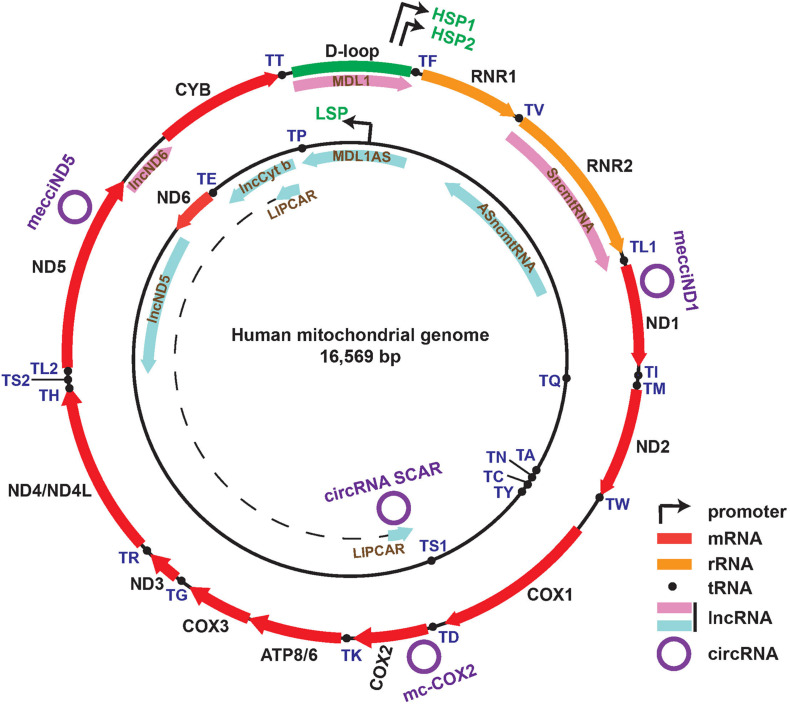
Map of the human mitochondrial genome with the following features: Genes corresponding to 13 mRNAs, 2 rRNAs, and 22 tRNAs within heavy (outer track) and light (inner track) strands; D-loop region and promoters (HSP1, HSP2, LSP) of both strands; eight mitochondria-encoded lncRNAs shown in the inner side of each ring (lnc*ND5*, lnc*Cyt b*, lnc*ND6*, MDL1S, MDL1AS, SmtncRNA, ASmtncRNA, and LIPCAR); four recently reported functional circRNAs (mecciND1, mecciND5, mc-COX2, circRNA SCAR).

Mitochondria have independent circular genomes (mtDNA) (Figure 1). Human mtDNA is mostly coding (more than 90%) and transcribed entirely in a bidirectional, polycistronic manner. Both strands of mtDNA harbor coding information. The heavy strand encodes 12 protein genes which are all subunits of the oxidative phosphorylation system (OXPHOS), two ribosomal RNAs (12S and 16S), and 14 tRNAs. The light strand encodes for a single mRNA (ND6) and 8 tRNAs (Scarpulla, 2008; Gustafsson et al., 2016; Barshad et al., 2018). Transcription is initiated from two heavy-strand promoters (HSP1, HSP2) and one light-strand promoter (LSP), located in the major non-coding region named “D-loop”. Two long polycistronic RNAs produced from HSP2 and LSP cover almost the full-length of each strand and another transcript transcribed from HSP1 consists of the two rRNA genes (Taanman, 1999; Asin-Cayuela and Gustafsson, 2007). The long mitochondrial precursor transcripts undergo the generally accepted “tRNA punctuations” processing by RNase P and RNase Z (ELAC2) cleavage to generate individual mRNAs, rRNAs, and tRNAs (Ojala et al., 1981; Holzmann et al., 2008; Lopez Sanchez et al., 2011; Barchiesi and Vascotto, 2019).

Recent years have witnessed the fast growing in understanding the great complexity of transcripts from the nuclear genome (Djebali et al., 2012; Iyer et al., 2015), and growing evidence has also demonstrated the presence of mitochondria-encoded non-coding RNAs (ncRNAs) such as long non-coding RNA (lncRNA) (Jusic and Devaux, 2020), circular RNAs (circRNAs) (Liu et al., 2020; Wu et al., 2020; Zhao et al., 2020), small non-coding RNAs (sncRNAs) (Mercer et al., 2011; Jusic and Devaux, 2020), and double-stranded RNAs (dsRNAs) (Dhir et al., 2018) with potential regulatory functions, respectively (Figure 1).

In this review we will focus on known regulatory non-coding RNAs encoded by the mitochondrial genome, and we also present a summarization of nuclear-encoded non-coding RNAs found in mitochondria for overall understandings about ncRNAs in mitochondria-nucleus communication. We highlight and discuss about their functional relevance in biomedicine, and in the context of cross-talk between mitochondria and the nucleus.

## Mitochondria-Encoded lncRNAs

**FIGURE 2:**
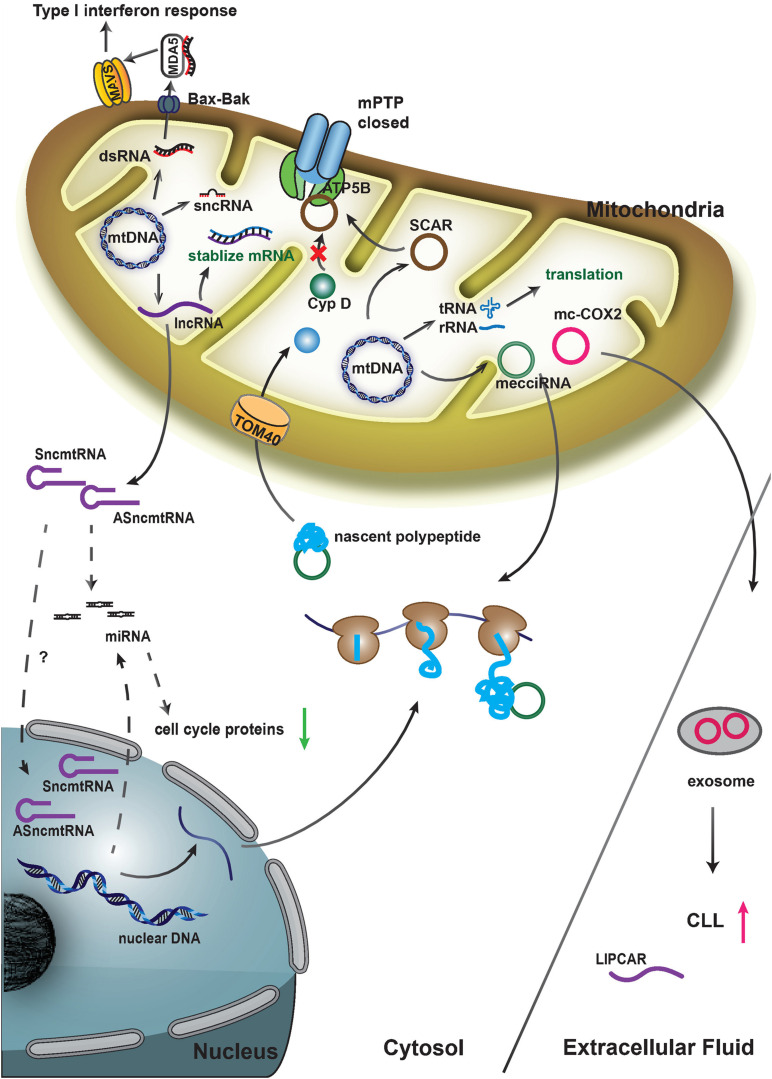
Proposed functions and functional mechanisms of mitochondria-encoded noncoding RNAs.

LncRNA is a major class of non-coding RNAs that is widely expressed in cells. Nuclear-encoded lncRNAs play diverse regulatory roles in mammalian cells, including transcriptional regulation, translational regulation, protein scaffolding, chromosome remodeling etc. (Gomes et al., 2013; Rashid et al., 2016; Marchese et al., 2017; Chen and Shan, 2020). A series of lncRNAs transcribed from the mtDNA have been recently reported, and these lncRNAs may also participate in multiple biological processes (Figure 2 and Table 1).

**TABLE 1:** Mitochondria-encode non-coding RNAs.

mt-ncRNAs	Type	Distribution	Proposed function	References
tRNAs	tRNA	Mitochondria	Mitochondrial mRNA translation	Taanman, 1999
16S rRNA 12S rRNA	rRNA	Mitochondria	Components of mitochondrial ribosome	Taanman, 1999
lnc*ND5*, lnc*Cyt b*, lnc*ND6*	lncRNA	Mitochondria	Stabilization of ND5, ND6, and Cyt b mRNAs through forming RNA-RNA duplexes	Rackham et al., 2011
SncmtRNA, ASncmtRNAs	lncRNA	Ubiquitous	Cell-cycle regulation in cancer cells	Villegas et al., 2007; Burzio et al., 2009; Landerer et al., 2011; Fitzpatrick et al., 2019; Lobos-González et al., 2020; Borgna et al., 2020
LIPCAR	lncRNA	Plasma	A risk-free biomarker for cardiac remodeling	Kumarswamy et al., 2014; Yang et al., 2014;
MDL1, MDL1AS	lncRNA	Mitochondria	Precursors of some tiRNAs	Gao et al., 2018; Xu et al., 2019
dsRNAs	dsRNA	Mitochondria Cytosol	Participate in the activation of innate immune defenses through MDA5–MAVS antiviral signaling axis	Dhir et al., 2018; Wiatrek et al., 2019
mecciND1, mecciND5	circRNA	Mitochondria Cytosol	Promote mitochondrial protein import	Liu et al., 2020
mc-COX2	circRNA	Exosome	Promotes CLL cell proliferation	Wu et al., 2020
circRNA SCAR	circRNA	Mitochondria	Binds to ATP5B and inhibits mitochondrial ROS output and fibroblast activation	Zhao et al., 2020
mitosRNAs	sRNA	Mitochondria	Might be involved in the regulation of normal expression of mitochondrial genes	Mercer et al., 2011; Ro et al., 2013
hsa-miR-4461, hsa-miR-4463, hsa-miR-4484, hsa-miR-4485	sRNA	Mitochondria	May regulate site-specific turnover of target mRNAs	Sripada et al., 2012
hsa-tir-MDL1AS-18	sRNA	Mitochondria	Highly expressed in normal tissues than in Hepatocellular Carcinoma (HCC) tissues	Gao et al., 2018
piRNAs	sRNA	Mitochondria	Unknown	Esteller, 2011; Larriba et al., 2018

Three lncRNAs (lnc*ND5*, lnc*Cyt b*, lnc*ND6*) generated from the mitochondrial genome were identified by Rackham et al. (2011) from deep sequencing data of human HeLa mitochondria (Rackham et al., 2011). The positions of these three lncRNAs corresponded to the regions complementary to the mitochondrial ND5, ND6, and Cyt b genes. There was no significant open reading frame present in them. It was shown that the three lncRNAs might play a functional role to stabilize ND5, ND6, and Cyt b mRNAs or to regulate the expression of the three genes in mitochondria through forming RNA-RNA duplexes (Rackham et al., 2011). The abundance of the three lncRNAs was cell and tissue-specific, indicating that they might be subjected to cell specific regulation and to play physiological roles.

Villegas et al. (2007) reported a 2,374 nt human mitochondrial transcript containing an inverted repeat (IR) of 820 nt linked to the 5′ end of the 16S mt-rRNA and named it SncmtRNA (sense non-coding mitochondrial RNA) (Villegas et al., 2007). In SncmtRNA, the IR generates a stem-loop structure with a double stranded region and a 40 nt loop. SncmtRNA was over-expressed in several tumor cell lines but not in resting cells (Villegas et al., 2007). The same researcher team later identified 2 transcripts containing IRs linked to the 5′ region of the antisense transcript of 16S mt-rRNA gene in the L-strand of the mtDNA, and designated them antisense ncmtRNA-1 (ASncmtRNA-1, 310 nt IR) and antisense ncmtRNA-2 (ASncmtRNA-2, 545 nt IR) (Burzio et al., 2009). AsncmtRNAs were expressed in normal proliferating cells but were down-regulated in different types of human tumor cells (Burzio et al., 2009). The subcellular location of SncmtRNA and ASncmtRNAs seemed to be nucleus (Landerer et al., 2011). Knockdown of ASncmtRNAs (ASK for short) by antisense oligonucleotide (ASO-1537S) targeting the single-stranded loop region resulted in Dicer-mediated release of hsa-miR-4485. hsa-miR-4485 in combination with nuclear miRNAs (mainly hsa-miR-5096 and hsa-miR-3609), which were induced by ASK, inhibited translation of 5 important cell cycle proteins (Cyclin B1, Cyclin D1, CDK1, CDK4, Survivin), and thereby induced growth inhibition of breast cancer cells (Fitzpatrick et al., 2019). The same researcher group later reported that exosomes derived from ASO-1537S-treated MDA-MB-231 breast cancer cells inhibited tumorigenesis of recipient cells (Lobos-González et al., 2020). And they also observed ASK induced bladder cancer cell death and inhibition of tumor growth (Borgna et al., 2020). The above results indicated that ASncmtRNAs could be potential therapeutic targets for breast cancer and bladder cancer.

Global transcriptomic profiling of human left ventricular (LV) from heart failing patients and controls revealed that in LV, relatively high abundance (71%) of lncRNAs were encoded by mitochondrial genome (mito-lncRNAs), and the abundance of mito-lncRNAs showed some but not statistically significant reduction in both non-ischemic and ischemic human failing hearts (Yang et al., 2014). A mitochondria-encoded chimeric transcript, long intergenic non-coding RNA predicting cardiac remodeling (LIPCAR) with the 5′-half (1–392 nt) mapped to lnc*Cyt b* and 3′-half (385–781 nt) mapped to antisense of mt-COX2 gene was identified in plasma from myocardial infarction patients (Kumarswamy et al., 2014). The expression level of LIPCAR was downregulated at the early stage of myocardial infarction but upregulated during later stages; in addition, high LIPCAR levels were associated with future cardiovascular death. The circulating LIPCAR has a potential to be a risk-free biomarker of LV remodeling and a predictor of patient survival of heart failure (Kumarswamy et al., 2014).

Using a PacBio full-length third-generation sequencing transcriptome dataset, Gao et al. (2018) discovered two polycistronic transcripts hsa-MDL1 (Mitochondrial D-loop 1) and hsa-MDL1AS (Mitochondrial D-loop 1 antisense) generated from the region covering the tRNA^Pro^ gene and full length of the human D-loop region (Gao et al., 2018). The presence of these transcripts was further confirmed by pan RNA-seq analysis (Xu et al., 2019). The mature hsa-MDL1 was generated from the H-strand polycistronic transcript, and hsa-MDLAS was antisense to hsa-MDL1 with much lower expression level. A great number of small RNAs reversely aligned to the mitochondrial D-loop region and a relatively lower abundant forwardly aligned small RNA (5% of reversely aligned ones) were obtained by searching public small RNA-sequencing data. hsa-MDL1 and hsa-MDL1AS were believed to be precursors of some of these small RNAs (Gao et al., 2018). Pan RNA-seq analysis also revealed the ubiquitous existence of 5′ and 3′ end small RNAs of MDL1 and MDL1AS (Xu et al., 2019).

## Mitochondria-Encoded dsRNAs

Dhir et al. (2018) discovered highly unstable mitochondrial double-stranded RNA (mt-dsRNA) in HeLa cells, which was supposed to be the natural outcomes of the nearly complete transcription of both heavy and light strands of mtDNA. It has been known that almost the entire L-strand transcript undergoes rapid RNA turnover by the mitochondrial RNA degradosome (Borowski et al., 2013), and indeed the level of mt-dsRNAs was restricted by the RNA degradosome components SUV3 and PNPT1 (Dhir et al., 2018). Pathological PNPT1 mutations led to abnormal mt-dsRNA accumulation. The Bax-Bak dependent release of mt-dsRNAs into cytoplasm triggered the upregulation of interferon-stimulated genes and the activation of innate immune defenses through the MDA5–MAVS axis (Dhir et al., 2018). In Trp53 mutant mouse embryonic fibroblasts, mitochondrial dsRNAs cleaved by RNase L were immunogenic, and could activate the type I interferon (IFN) pathway via RIG-I-like receptors (Wiatrek et al., 2019).

## Mitochondria-Encoded circRNAs

circRNAs encoded by mammalian mitochondrial genome had not been revealed until recent report about the identification of hundreds of mitochondria encoded circRNAs (mecciRNAs) in human and murine cells through second-generation sequencing of mitochondrial RNAs (Liu et al., 2020, 2021). In three cell lines and one tumor tissue (hepatocellular carcinoma) from human and two cell lines together with two tissues (skeletal muscle and heart) from mice, 248 human mecciRNAs and 268 murine mecciRNAs were identified, respectively (Liu et al., 2020). Actually, the presence of large amount of mecciRNAs was not mammalian specific, as over one hundred mecciRNAs were also identified in zebrafish (Liu et al., 2020). mecciRNAs were encoded by both the light and heavy strands of the mtDNA, although the heavy strand of mtDNA encoded the majority (Liu et al., 2020). The presence of mitochondria encoded circRNAs in human HEK293 cells was examined by RT-PCR assay to amplify over one hundred individual mecciRNAs (Mance et al., 2020). With third-generation sequencing, mecciRNAs from murine brain were also analyzed for their junctions and full length (Zhang et al., 2021).

It was found that some mecciRNAs distributed both inside the mitochondria and outside in the cytosol (Liu et al., 2020). Functional studies of two mecciRNAs, mecciND1 (encoded by the mitochondrial ND1 gene) and mecciND5 (encoded by the mitochondrial ND5 gene) demonstrated that these mecciRNAs conducted essential physiological functions. mecciND1 bound to RPA1 and RPA2 proteins, which were involved in mtDNA replication. The expression level of mecciND1 was positively related to mitochondrial RPA protein levels and mtDNA copy numbers. mtDNA copy numbers were increased after mecciND1 overexpression and decreased under mecciND1 knockdown. mecciND5 interacted with hnRNPA1, hnRNPA2B1 and hnRNPA3 and promoted their mitochondrial importation (Liu et al., 2020). Both *in vivo* and *in vitro* evidence showed that the two mecciRNAs, mecciND1 and mecciND5, could interact with TOM40 and PNPASE, and function as molecular chaperons to facilitate the mitochondrial entry of newly synthesized polypeptides encoded by the nuclear genome (Liu et al., 2020; Figure 2). mecciND1 and meccciND5 were upregulated in hepatocellular carcinoma (HCC) tissues, and moreover, mecciRNA levels were regulated in stress conditions, suggesting that they were critical for cells to cope with physiological and pathological changes (Liu et al., 2020).

When investigating the role of mitochondria-located circRNAs in metaflammation, Zhao et al. (2020) observed that 3 mitochondria-encoded circRNAs were downregulated in liver fibroblasts from patients with non-alcoholic steatohepatitis (NASH). One of the three mecciRNAs, termed SCAR (Steatohepatitis-associated circRNA ATP5B Regulator), was an antisense RNA from the locus COX2. SCAR bound directly to ATP5B, a mitochondrial permeability transition pore (mPTP) regulator. The interaction of ATP5B and SCAR shut down mPTP by blocking Cyclophilin D-mPTP interaction, and therefore, inhibited mitochondrial ROS (mROS) output (Zhao et al., 2020; Figure 2). mROS output was required for liver fibroblast activation, and this circRNA had a potential to serve as a therapeutic target for NASH (Zhao et al., 2020). Another highly expressed mecciRNA, mc-COX2, a sense RNA from the locus COX2, was found in the plasma exosomes of chronic lymphocytic leukemia (CLL) patients (Wu et al., 2020). mc-COX2 was closely correlated to prognosis of CLL, and moreover, it seemed that higher expression levels of mc-COX2 could promote cell proliferation and protect cells from apoptosis (Wu et al., 2020; Figure 2).

## Mitochondria-Encoded sncRNAs

In mammalian cells, small non-coding RNAs (sncRNAs) are diverse and abundant, mainly including microRNAs (miRNAs), endogenous-short interfering RNA (siRNAs), PIWI-interacting RNAs (piRNAs), and other types of small RNAs derived from tRNAs, rRNAs, and snoRNAs (Gomes et al., 2013). Recent studies have identified several types of sncRNAs that are generated from mammalian mitochondrial genome (Table 1).

Mercer et al. (2011) provided a comprehensive description of the human mitochondrial transcriptome. They sequenced mitochondrial small RNAs (sRNAs) isolated from human bone osteosarcoma cell line 143B and revealed 31 sRNAs of two distinct classes of 21 and 26 nt from 17 loci of mtDNA. The majority (84%) of them were produced from tRNA genes. Expression of these mitochondria-encoded sncRNAs changed dynamically in different cell types, however, the expression of sncRNAs did not seem to correlate significantly with the expression of overlapping genes (Mercer et al., 2011). Ro et al. (2013) reported that thousands of small RNAs were encoded from murine and human mitochondrial genomes, and major size of these sRNAs ranged between 30 and 39 nt (mouse) and 20 and 29 nt (human). These RNAs were mainly transcribed from the sense direction of the mitochondrial genes (host genes), and only a small portion were from the antisense direction of their host genes. Those sRNAs appeared to target antisense transcripts and promoted the expression of their host genes *in vitro* (Ro et al., 2013). Interestingly, all these researches provided evidence that these mitochondria-encoded sncRNAs were generated in a Dicer-independent manner, and might be products of some unknown ribonucleases within the mitochondria (Mercer et al., 2011; Ro et al., 2013).

In human HEK293 and HeLa mitochondria, 4 known miRNAs (hsa-miR-4461, hsa-miR-4463, hsa-miR-4484, and hsa-miR-4485) and 24 putative novel miRNAs could be aligned to mitochondrial genome at the positions corresponding to 16S rRNA, tRNA, and mRNA (Sripada et al., 2012). These miRNAs might regulate site-specific turnover of target mRNAs. However, it was not conclusive that whether these microRNAs were actually transcribed from mitochondrial genome (Sripada et al., 2012). A series of small RNAs that aligned to the D-loop region of mtDNA were discovered by Gao et al. (2018). These small RNAs might be generated from mitochondrial lncRNAs hsa-MDL1AS and hsa-MDL1. The most abundant small RNA was hsa-tir-MDL1AS-18, derived from lncRNA hsa-MDL1AS. It belonged to transcription initial RNAs (tiRNAs), as 18 nucleotides from its 5′ end precisely overlapped with transcription initiation (IT) sites of the L-strand promoter. hsa-tir-MDL1AS-18 was down-regulated in hepatocellular carcinoma (HCC) tissues, suggesting that the balance of tiRNAs/lncRNAs regulation might be abnormal in cancer cells (Gao et al., 2018).

Through mapping known piRNA sequences to the human mtDNA, Kwon et al. (2014) identified 29 piRNAs, and 12 out of these 29 piRNAs matched to the stem-loop fragments of seven mitochondrial tRNAs with asymmetric tRNA fragment usage and cell type specific expression. They also reported the presence of PIWI through Western blots and three abundant mature piRNAs by RT-PCR in the mitochondria of HeLa-S3 cells (Kwon et al., 2014). Mitochondria-encoded piRNAs might also be present in mouse (Larriba et al., 2018). Among all the small RNA reads from oocytes and zygotes of mice, about 20% of the reads were mapped to mitochondrial DNA, and 80–90% of the reads were classified as piRNAs based on the size; these piRNAs were encoded by both strands of the mouse mitochondrial genome, and were highly expressed from the loci corresponding to mitochondrial tRNAs, 16S rRNA, and D-loop region (Larriba et al., 2018). Functional study of mitochondrial piRNAs is lacking, and these piRNAs are speculated to function in stress responses (Kwon et al., 2014), gamete differentiation, and fertilization (Larriba et al., 2018).

## Nuclear-Encoded ncRNAs in Mitochondria

In mammalian mitochondria, besides some well-known nuclear encoded tRNAs (tRNA^Leu^UAA, tRNA^Gln^UUG, and tRNA^Gln^CUG) (Rubio et al., 2008; Mercer et al., 2011; Gowher et al., 2013), 5S rRNA (Yoshionari et al., 1994; Magalhães et al., 1998; Entelis et al., 2001; Mercer et al., 2011; Zelenka et al., 2014; Autour et al., 2018), RMRP RNA (Chang and Clayton, 1987; Li et al., 1994; Wang et al., 2010; Noh et al., 2016), some nuclear-encoded ncRNAs including miRNAs and lncRNAs were also found to play critical roles in mitochondria (Kim et al., 2017; Jeandard et al., 2019; Table 2). miRNAs were described in purified mitochondria of rat and mouse through miRNA microarray analysis (Kren et al., 2009; Bian et al., 2010). Bandiera et al. (2011) identified 13 nuclear-encoded miRNAs enriched in mitochondria of HeLa cells, and defined these mitochondria-located miRNAs as mitomiRs. Meanwhile, Barrey et al. (2011) predicted 25 pre-miRNAs and 33 miRNAs in human mitochondrial *in silico* sequencing analysis, and a set of them were demonstrated to be present in the mitochondria of skeletal muscular cells by *in situ* hybridization and RT-PCR (Barrey et al., 2011). Moreover, both studies revealed that these miRNAs might post-transcriptionally regulate mt-RNAs in mitochondria through RNA interfering pathway (Bandiera et al., 2011; Barrey et al., 2011). Nuclear DNA-encoded miR-18c was found to be translocated into the mitochondria of rat cardiac myocytes (Das et al., 2012). miR-18c loaded in AGO2n bound to the 3′ UTR of mt-COX1, and inhibited mt-COX1 translation, ultimately leading to complex IV remodeling and mitochondrial dysfunction (Das et al., 2012). mitomiR-378 conducted a similar function that downregulated mt-ATP6 translation in mouse HL-1 cells (Jagannathan et al., 2015). Fan and co-workers identified mitomiR-2392 was upregulated in cisplatin-resistant tongue squamous cell carcinoma (TSCC) cells and TSCC tumors (Fan et al., 2019). Surprisingly, mitomiR-2392 was found to partially repress the transcription of mtDNA rather than the translation, through miRNA:mtDNA base pairing with an AGO2-dependent manner (Fan et al., 2019). The downstream genes regulated by mitomiR-2392 were different between two TSCC cell lines, suggesting a cell-specific regulation (Fan et al., 2019). In addition to inhibitory effects, some mitomiRs were found playing positive regulatory functions. For example, miR-1 was induced and imported into mitochondria during myogenesis to stimulate the translation of mt-ND1 and mt-COX1, through specific miRNA:mRNA base-pairing with the participation of AGO2 in mouse heart and C2C12 cells (Zhang et al., 2014). Most mitomiRs with demonstrated functions played AGO2-dependent roles, and an array of evidence showed that AGO2 was present in mitochondria (Bandiera et al., 2011, 2013; Zhang et al., 2014; Vendramin et al., 2017).

**TABLE 2:** Nuclear-encoded non-coding RNAs in mitochondria.

ncRNAs	Type	Function in cytosol/nucleus	Proposed function in mitochondria	References
tRNAs (tRNA^Leu^UAA, tRNA^Gln^UUG, tRNA^Gln^CUG)	tRNA	Translation	May participate in translation	Rubio et al., 2008; Gowher et al., 2013
Various miRNAs (hsa-miR-494, hsa-miR-1275, hsa-miR-1974, miR18c, miR-378 etc.) and pre-miRNAs	miRNA	RNA interfering pathway	Translation inhibition	Bandiera et al., 2011; Barrey et al., 2011; Das et al., 2012; Jagannathan et al., 2015
mitomiR-2392	miRNA	RNA interfering pathway	Transcription repression	Fan et al., 2019
miR-1	miRNA	RNA interfering pathway	Translation activation	Zhang et al., 2014
5S rRNA	rRNA	Component of the cytosolic ribosome	May participate in translation	Yoshionari et al., 1994; Magalhães et al., 1998; Entelis et al., 2001; Autour et al., 2018; Mercer et al., 2011; Zelenka et al., 2014
RMRP	lncRNA	5.8S rRNA processing	Mitochondrial RNA processing	Chang and Clayton, 1987; Li et al., 1994; Wang et al., 2010; Noh et al., 2016
SAMMSON	lncRNA	Interacts with p32 and facilitates its targeting to the mitochondria	Unknown	Leucci et al., 2016; Vendramin et al., 2018
H1 RNA	lncRNA	RNA component of nuclear RNase P	May participate in mitochondrial RNA processing	Bartkiewicz et al., 1989; Puranam and Attardi, 2001
hTERC	lncRNA	RNA component of Telomerase	May be processed in mitochondria and transported back to cytosol	Cheng et al., 2018

The malignant lncRNA SAMMSON (survival associated mitochondrial melanoma specific oncogenic non-coding RNA) was predominantly localized in the cytoplasm of human melanoblasts and melanoma cells, and interestingly, a large fraction of cytoplasmic SAMMSON was found to co-localize and co-purify with mitochondria (Leucci et al., 2016). SAMMSON played pro-oncogenic roles in mitochondrial homeostasis and metabolism by interacting with and stabilizing p32 protein, a regulator of the maturation of mitochondrial 16S rRNA (Leucci et al., 2016; Vendramin et al., 2018). Human nuclear RNase P RNA component (H1 RNA) was reported in mitochondria of HeLa cells (Bartkiewicz et al., 1989; Puranam and Attardi, 2001). However, as human mitochondrial RNase P was a protein-only complex and a trans-acting RNA component was not required for catalysis (Holzmann et al., 2008), the role of H1 RNA in mitochondria was still unclear. Cheng et al. (2018) found that human telomerase RNA component (hTERC) was imported into mitochondria, processed to a shorter form hTERC-53, and then exported back to the cytosol (Cheng et al., 2018). The underlaying mechanism and cellular significance of this cytosol and mitochondria import-export process of hTERC-hTERC-53 is elusive and needs further investigation.

## Discussion

It is apparent that the known complexity of mitochondrial transcriptome of human and mammals has been greatly expanded. Mitochondrial genome encodes not only very limited numbers of mRNAs, rRNAs and tRNAs as previously understood, but also a variety of non-coding RNAs such as lncRNAs, circRNAs, sncRNAs, and dsRNAs with diverse regulatory functions. Mitochondria seem to have sacrificed the protein coding ability, although maintained the capacity of generating their “own” complex ncRNA profiles, with the small size but highly utilized genome.

Mitochondria-encoded lncRNAs seem function through RNA-RNA interaction or as precursors of small RNAs to regulate the stabilization or expression of their corresponding mt-RNAs in mitochondria (Rackham et al., 2011; Gao et al., 2018). Several mt-lncRNAs can be detected outside the mitochondria, such as LIPCAR in plasma, SncmtRNA and ASncmtRNAs in nucleus, but the mechanisms of their transport or function are still elusive (Landerer et al., 2011; Kumarswamy et al., 2014). circRNAs encoded by the mammalian nuclear genome are known to function as microRNA sponges, transcriptional regulators, protein binding partners, and templates for protein translation (Chen et al., 2015; Li et al., 2015; Kristensen et al., 2019; Chen and Shan, 2021). Mitochondria-encoded circRNAs may reside in or shuttle in and out of the mitochondria to play critical roles, and the functions and functional mechanisms of mecciRNAs are waiting for further in-depth investigation (Liu et al., 2020; Zhao et al., 2020). The sRNAs, miRNAs, piRNAs, and other snRNAs encoded by mitochondrial genome are generally classified by their size, and the functions of these non-coding RNAs need further elucidation. Some nuclear genome encoded miRNAs (mitomiRs) can also function in mitochondria. Studies of these mitomiRs may provide some implications for future investigation on mitochondria-encoded sncRNAs. The presence of AGO2 in mitochondria makes it reasonable to speculate that certain sncRNAs can also function through RNAi machinery in mitochondria (Bandiera et al., 2011; Bandiera et al., 2013; Zhang et al., 2014; Vendramin et al., 2017). The functional characterizations of mitochondria-encoded ncRNAs are just emerging, and the biogenesis and metabolism of mitochondria-encoded ncRNAs, including circRNAs, lncRNAs, and sncRNAs, remain largely obscure.

Due to the limited coding capacity of mitochondrial genome, it is known that a large number of proteins encoded by the nuclear genome are imported into mitochondria from cytosol to sustain the biogenesis and function of these organelles (Harbauer et al., 2014; Wiedemann and Pfanner, 2017). Mitochondria generate energy, ROS, and other metabolic molecules for multiple essential cellular events, and also participate in multiple pathways regulating nuclear gene expression, cell death, proliferation, differentiation, etc. (McBride et al., 2006). The growing evidence and examples reviewed in this article have shown that non-coding RNAs are engaged in the bidirectional communication between mitochondria and the nucleus (Vendramin et al., 2017; Gusic and Prokisch, 2020). Nuclear DNA-encoded miRNAs and lncRNAs can also be transported into mitochondria and regulate mitochondrial gene expression transcriptionally and post-transcriptionally. However, the import mechanisms are still poorly understood. Several lines of evidence show that tRNAs are imported into mitochondria by an ATP dependent mechanism that is distinct from protein import (Rubio et al., 2008; Gowher et al., 2013). Disruption of the mitochondrial membrane potential, which is crucial for protein import, has no effect on tRNA import (Rubio et al., 2008). There is only one protein PNPT1 (also named PNPASE), which locates in the intermembrane space (IMS) of mitochondria, known to facilitate the transport of RMRP, H1 RNA, and hTERC through binding to small stem-loops of these RNAs (Wang et al., 2010; Kim et al., 2017; Jeandard et al., 2019). Mitochondria-encoded ncRNAs contribute to the nucleus-mitochondria communication, and some of them not only function inside the mitochondria but also play roles outside of the mitochondria. For example, mecciRNAs shuttle between mitochondria and cytosol to facilitate the mitochondrial entry of newly synthesized polypeptides encoded by the nuclear genome (Liu et al., 2020). ASncmtRNAs are reported with nuclear and cytosolic localization, and regulate the translation of cell cycle proteins (Landerer et al., 2011; Fitzpatrick et al., 2019). But how these ncRNAs get transported through the bilayer membrane of mitochondria is still unclear, and further investigations about the mechanisms will contribute to a deeper understanding of mitochondria-nucleus communication.

Mitochondrial dysfunction relates to a series of diseases, such as cardiovascular diseases, cancers, and neurodegeneration (Herst et al., 2017; Jusic and Devaux, 2020). The cell/tissue specific expression of mt-ncRNAs also suggests that they may take part in mitochondria-related diseases. The investigations of mitochondrial ncRNAs regulatory network would contribute to better understanding of etiology, and lead to novel diagnostic and therapeutic approaches for mitochondrial dysfunction-related diseases. Mitochondria-encoded non-coding RNAs have no doubt pointed to a new direction for the study of mitochondria-nucleus communication, and study about these RNAs is an important field of biomedicine.

## Author Contributions

GS and XL conceived the scope of the manuscript and wrote the manuscript. Both authors contributed to the article and approved the submitted version.

## Conflict of Interest

The authors declare that the research was conducted in the absence of any commercial or financial relationships that could be construed as a potential conflict of interest.

## Publisher’s Note

All claims expressed in this article are solely those of the authors and do not necessarily represent those of their affiliated organizations, or those of the publisher, the editors and the reviewers. Any product that may be evaluated in this article, or claim that may be made by its manufacturer, is not guaranteed or endorsed by the publisher.
